# Do microbes have a memory? History-dependent behavior in the adaptation to variable environments

**DOI:** 10.3389/fmicb.2022.1004488

**Published:** 2022-10-10

**Authors:** Lieselotte Vermeersch, Lloyd Cool, Anton Gorkovskiy, Karin Voordeckers, Tom Wenseleers, Kevin J. Verstrepen

**Affiliations:** ^1^VIB – KU Leuven Center for Microbiology, Leuven, Belgium; ^2^CMPG Laboratory of Genetics and Genomics, KU Leuven, Leuven, Belgium; ^3^Laboratory of Socioecology and Social Evolution, Department of Biology, KU Leuven, Leuven, Belgium

**Keywords:** history-dependent behavior, fluctuating environments, epigenetic mechanisms, cellular heterogeneity, *Saccharomyces cerevisiae*

## Abstract

Microbes are constantly confronted with changes and challenges in their environment. A proper response to these environmental cues is needed for optimal cellular functioning and fitness. Interestingly, past exposure to environmental cues can accelerate or boost the response when this condition returns, even in daughter cells that have not directly encountered the initial cue. Moreover, this behavior is mostly epigenetic and often goes hand in hand with strong heterogeneity in the strength and speed of the response between isogenic cells of the same population, which might function as a bet-hedging strategy. In this review, we discuss examples of history-dependent behavior (HDB) or “memory,” with a specific focus on HDB in fluctuating environments. In most examples discussed, the lag time before the response to an environmental change is used as an experimentally measurable proxy for HDB. We highlight different mechanisms already implicated in HDB, and by using HDB in fluctuating carbon conditions as a case study, we showcase how the metabolic state of a cell can be a key determining factor for HDB. Finally, we consider possible evolutionary causes and consequences of such HDB.

## Introduction: What is memory?

Cells are constantly faced with environmental changes. The most-studied response strategy to such a change is one of sense-and-respond, where cells induce an appropriate response through specific sensing-signaling pathways upon detecting a change. In principle, such sensing-signaling mechanisms seem ideal, as they enable cells to quickly launch a specific response. However, sustaining active sensing-signaling pathways requires expressing specific sensors and signaling pathway components, which comes at a fitness cost (Zaman et al., 2008; Govern and Ten Wolde, 2014). Moreover, in some cases, environmental changes are so abrupt or dramatic that they do not leave sufficient time for cells to launch a reaction. In these cases, other response strategies, such as stochastic switching, where cells randomly switch between different phenotypes, may provide a better solution because they imply that a fraction of the population is prepared for a change even before it occurs (Acar et al., 2008).

Alongside different response strategies, multiple studies have observed that in some cases, the response of cells can be influenced by previous exposure to environmental changes, and that this may even allow anticipating future changes. Examples of this complex phenomenon are both ubiquitous and diverse. A form of anticipatory behavior is observed in environments with a typical temporal order of events, where the presence of a specific condition serves as a cue for the likely arrival of another condition (Tagkopoulos et al., 2008; Mitchell et al., 2009). In such cases, cells may have evolved a signaling cascade that triggers an almost Pavlovian-like behavior where they respond to the first stimulus by already anticipating the next events that often follow. For example, Tagkopoulos et al. (2008) identified a possible anticipatory gene regulation pattern in *Escherichia coli*, where exposure to increased temperature also affects the expression of genes needed for growth under reduced oxygen availability. Interestingly, this specific order of events (increased temperature followed by lower oxygen levels) is also what *E. coli* cells experience when entering the warm, oxygen-deprived mammalian gut. Other examples of anticipatory behavior include adaptation of *E. coli* to the order in which they are exposed to different sugars in the digestive tract (Mitchell et al., 2009) as well as *Vibrio cholerae*’s ability to already induce genes required for life outside the host during the last stages of infection (Schild et al., 2007).

Apart from anticipatory behavior where cells anticipate that one specific change will be followed by another, exposure to a specific environmental condition can also accelerate or boost the response when that specific condition re-occurs. A well-known and highly complex example of such a response is adaptive immunity in vertebrates, where an initial response to a certain antigen results in an enhanced response to future encounters with that same antigen (Chaplin, 2010). Another form of such history-dependent behavior is also observed in microbes. Yeast cells, for example, are known to better respond to severe stress conditions when they have recently been exposed to similar but milder stress, a process sometimes referred to as “priming” (Guan et al., 2012; Brown et al., 2014; Skwark et al., 2017; Harish and Osherov, 2022).

**Figure 1:**
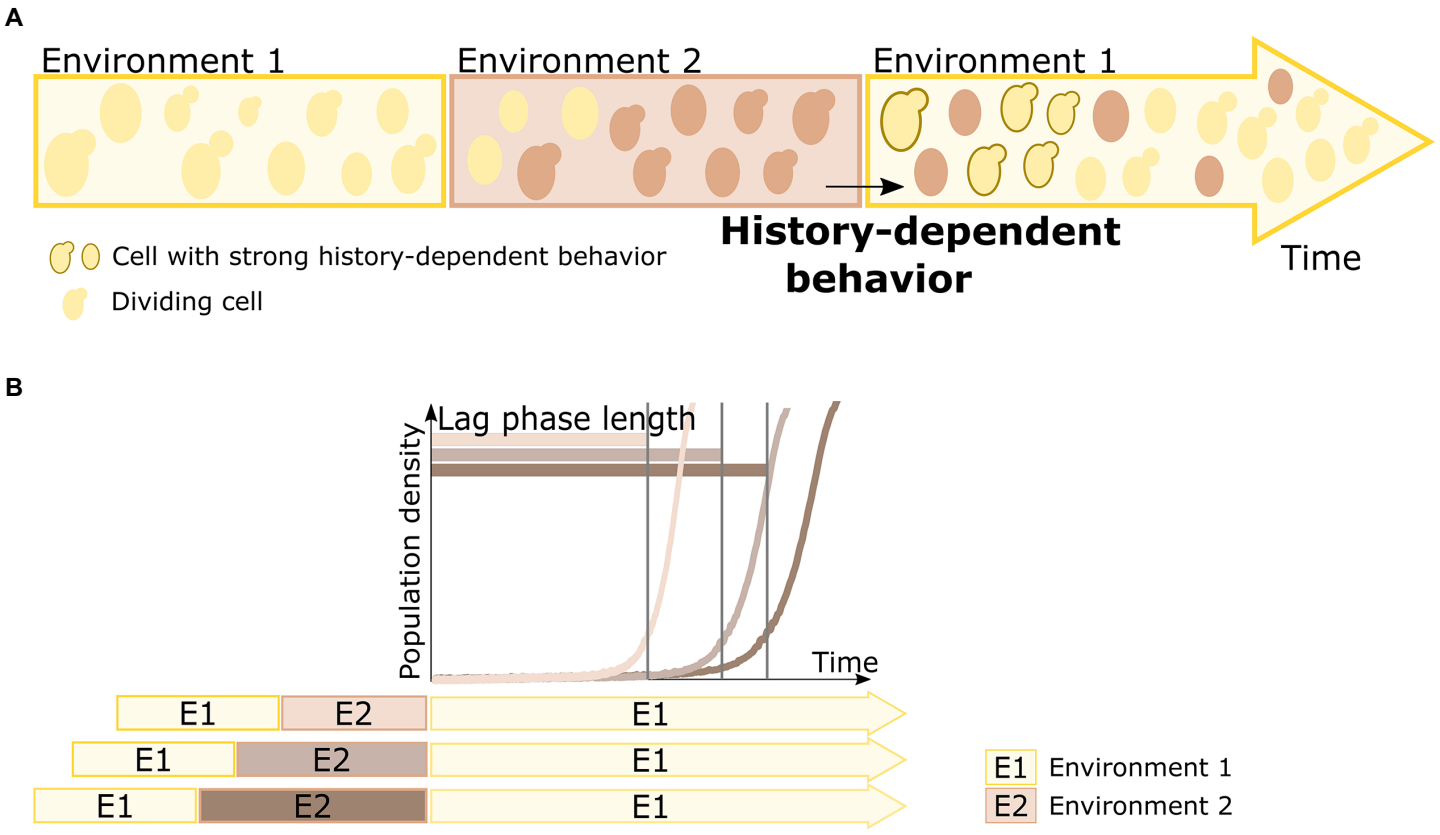
Principle of microbial history-dependent behavior. **(A)** A cell’s behavior can depend upon past experiences, a phenomenon termed history-dependent behavior. Cells encountering a specific environment need to adjust to that environment. Some cells are able to adapt faster based on their past experience, and thus display a strong history-dependent behavior. For example, when a population of cells encounters a specific environment (environment 1) again, there is a fraction of cells that can respond and adapt faster to this environment than others. These cells, indicated with a dark brown contour in the returning environment 1, display such history-dependent behavior. **(B)** Duration of the lag phase is an ideal read-out for history-dependent behavior in changing carbon environments. When shifted from one carbon source to another, yeast cells need to adapt their metabolism and express genes required for consumption of the specific carbon source present. This necessary adaptation often leads to a period of no or reduced growth (as visualized by a delay in increase of population density), called the lag phase. Not only the environmental change as such influences the lag phase length, but the lag phase length upon return of environment 1 (E1, indicated in yellow color) can also be influenced by how long ago the cells have been exposed to environment 1, with longer times spent in an intermediary environment 2 (E2, indicated in brown color) resulting in longer lag phases when the original environment 1 returns.

History-dependent behavior has also been observed in environments with alternating carbon sources (Figure 1A; Lambert and Kussell, 2014; New et al., 2014; Stockwell et al., 2015; Cerulus et al., 2018). When shifted from one carbon source to another, microbial cells need to adapt their metabolism and express genes required for consumption of the specific carbon source present. This necessary adaptation often leads to a period of no or reduced growth, called the lag phase (Jacob and Monod, 1961). Interestingly, the lag phase decreases when cells have encountered the previous carbon source in the past, indicating some kind of “memory” of those past environments (Figure 1B; Cerulus et al., 2018).

The term “cellular memory,” where a cell’s response depends upon its past experiences, has been widely used for a range of different phenomena with possibly quite different underlying mechanisms. To avoid any confusion, it is important to note that this cellular memory in microbes, as discussed in this review, is very different from neuronal memory, where past experiences are actively stored through complex neuronal synapses. Furthermore, throughout scientific literature, many different terms have been used to describe this cellular memory, including anticipatory behavior, phenotypic memory, response memory, hysteresis and history-dependent behavior (Mitchell et al., 2009; Lambert and Kussell, 2014; Cerulus et al., 2018). In this review, we will use the term “history-dependent behavior” (HDB) for any mechanism that makes an organism’s or a cell’s response depend upon past experiences. Moreover, cellular HDB can extend over several (cellular) generations and thus influence the behavior of progeny cells that have not directly experienced the initial condition. Specifically, we here define HDB as the behavior of an individual cell and its progeny based on the epigenetic memory of previous exposure(s) to particular environment(s). This HDB could benefit the population in the future. Below, we will focus specifically on mechanisms underlying microbial HDB in fluctuating environments, with a particular emphasis on HDB mechanisms in *Saccharomyces cerevisiae*. As will become clear from the examples discussed below, timescales associated with HDB can differ depending on the type of mechanism and environmental changes involved.

## Mechanisms underlying HDB

**Figure 2:**
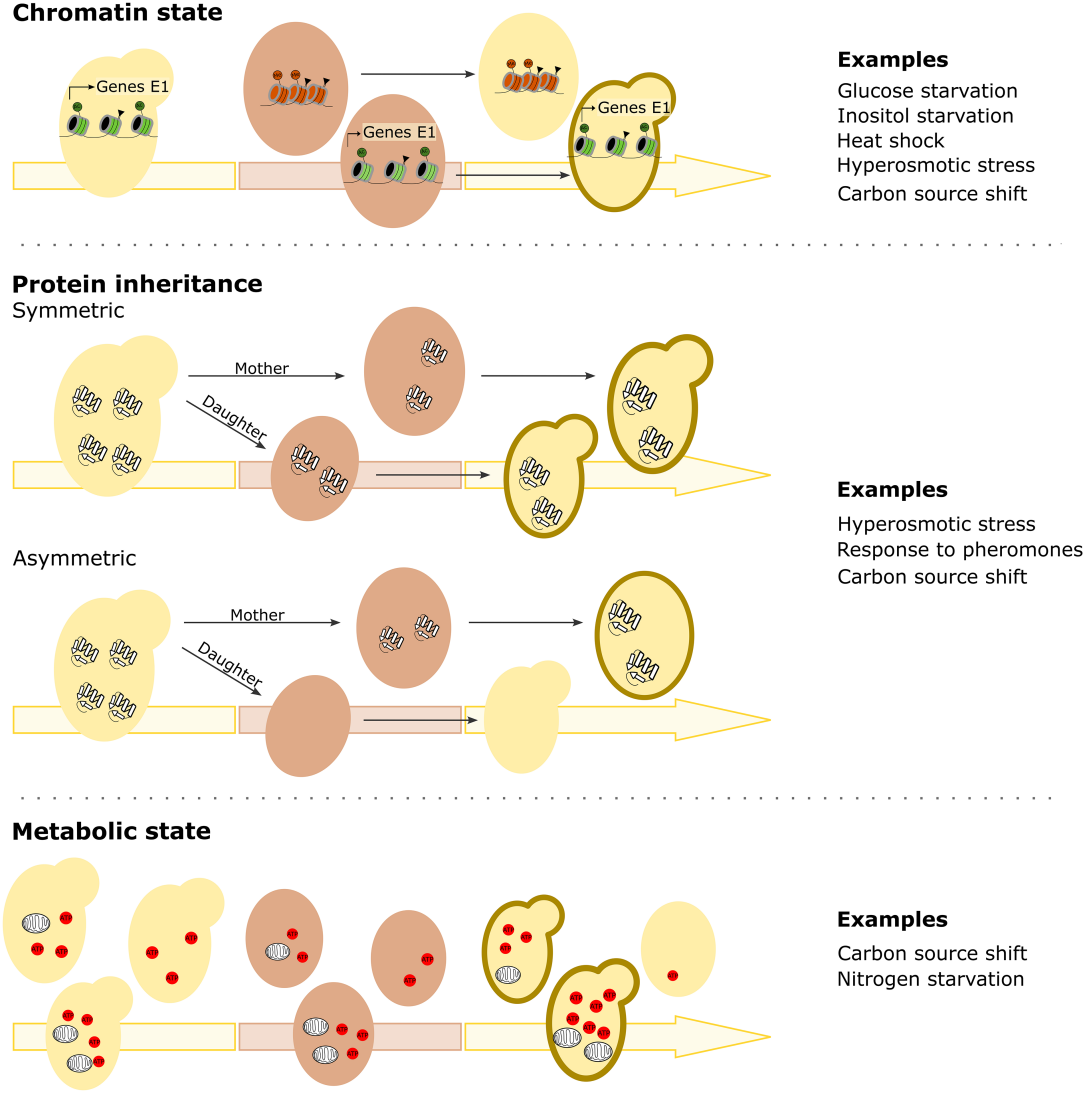
Mechanisms implicated in microbial history-dependent behavior. Chromatin state, protein inheritance and cellular metabolic state have all been described to underly history-dependent behavior. Semi-stable changes in *chromatin state* of promoters of specific genes can persist across environments, thus allowing cells to respond faster when that specific environment returns. Triangles indicate generic histone modifications, Ac (dark green circle) indicates acetylation and Me (dark orange circle) indicates methylation. In case of *protein inheritance*, proteins can remain stable and active over multiple cellular generations and/or across environments (*via* both symmetric and asymmetric protein inheritance). Also *cellular metabolic state* can play a role in history-dependent behavior, with for example a cell’s respiratory activity/capacity being a key factor of history-dependent behavior for carbon source changes. Figures depict the different mechanisms described, with examples of conditions in which this type of mechanism has been suggested to underly the observed history-dependent behavior. Cells indicated with a dark brown contour in the returning environment will show history-dependent behavior and thus be able to overcome the environmental change faster. For more details, see text.

While specific DNA changes determine and affect many cellular properties, it appears that epigenetic differences are key in determining how previous experiences can impact the (speed of) future cellular responses to a stimulus over time and even generations. The epigenetic mechanisms underlying HDB are diverse and likely multifaceted, with studies finding changes and differences in chromatin state, protein carryover, mitochondrial activity as well as specific biomolecules influencing HDB (Figure 2). Importantly, some forms of HDB may simply be consequences of the primary response mechanism and not be the result of adaptive evolution. However, since HDB may confer fitness benefits by optimizing a cell’s capacity to anticipate or respond to environmental changes, it seems likely that at least some forms of HDB may have been selected for. We will discuss this further in the section on evolutionary implications of history-dependent behavior.

### Chromatin state

A first mechanism that has been linked to HDB in yeast cells involves chromatin (Figure 2). It is well known that activating genes can sometimes require local chromatin remodeling to make the promoters of specific genes more accessible to the transcription machinery. Since this altered, open DNA structure is somewhat stable and heritable for a few cellular generations (Rando and Verstrepen, 2007), re-activation of the same genes upon a second exposure to similar conditions may happen more readily compared to a first exposure. Examples of such epigenetic transcriptional HDB have been described for inositol starvation, glucose starvation, nitrogen starvation as well as heat shock and hyperosmotic stress (Rienzo et al., 2015; D’Urso et al., 2016; Sood and Brickner, 2017; Ben Meriem et al., 2019; Sump et al., 2022; Wang et al., 2022).

Perhaps the best studied example of this chromatin-dependent HDB is the shift of *S. cerevisiae* cells between glucose and less preferred, so-called secondary carbon sources, like maltose or galactose. While a first shift from glucose to a secondary carbon source causes a relatively long lag phase, a second shift that occurs within a few cellular generations of a first transition often goes more smoothly, with cells resuming growth in less time (Kundu et al., 2007; Zacharioudakis et al., 2007; Cerulus et al., 2018; Perez-Samper et al., 2018). It is believed that the shorter lag phase is at least partly explained by semi-stable changes in the chromatin structure of genes encoding transporters and catabolic enzymes for non-preferred sugars. Indeed, exposure to these sugars induces decondensation of the respective promoters, possibly involving de-methylation of local nucleosomes (Tan-Wong et al., 2009; Brickner, 2010; Stockwell et al., 2015). This allows cells to respond and activate their metabolism faster when the non-preferred carbon source returns (Kundu et al., 2007). Factors having robust effects on this type of HDB include the SWI/SNF chromatin remodeling complex and the SET3 histone deacetylase complex (Kundu et al., 2007; Kim et al., 2012; Rienzo et al., 2015; Cerulus et al., 2018; Bheda et al., 2020).

### Protein inheritance

Another mechanism implicated in establishing HDB is protein carryover or protein inheritance (Figure 2). Since proteins can remain stable and active over multiple cellular generations, this mechanism can also help explain epigenetic inheritance of HDB. In the case of hyperosmotic stress for example, the buildup of protective proteins upon the first exposure to high salt concentrations can prepare the cells for a second occurrence of hyperosmotic shock and allow them to survive (Rienzo et al., 2015; Ben Meriem et al., 2019). Along those same lines, for yeast cells transitioning from glucose to galactose, Gal proteins were proposed to accumulate during galactose growth and be inherited from mother to daughter cells, persisting in the cytoplasm for multiple generations, even when galactose is no longer present in the environment. These Gal proteins only gradually disappear because of natural degradation and dilution during cell division, thus giving cells that still have a basal level of Gal proteins an advantage when the galactose environment returns, in terms of both energy and protein production and having a shorter lag phase (Zacharioudakis et al., 2007; Stockwell et al., 2015). Interestingly, the level of HDB in the shift from glucose to galactose differs between yeast species (Sood and Brickner, 2017). Galactose HDB in *S. cerevisiae* appears to at least partly depend on carryover of cytoplasmic Gal proteins such as the Gal1 galactokinase, which is strongly repressed during growth in glucose. On the other hand, in *S. uvarum*, basal expression levels of *GAL1* in glucose are higher, resulting in faster activation of *GAL* genes even at the first exposure to galactose, albeit at the cost of producing Gal proteins that are likely a useless metabolic burden during growth on glucose (Sood and Brickner, 2017).

Another example of HDB where protein levels play a central role is that of “deceptive courtship” in haploid yeast cells (Caudron and Barral, 2013). Upon detection of pheromones of a cell of the opposite mating type, yeast cells arrest their cell cycle and develop a so-called shmoo, a cytoplasmic projection towards the detected pheromone and thus mating partner (Jenness et al., 1983). However, if no partner can be reached within a reasonable time, cells will escape the pheromone-induced cell cycle arrest and divide (Moore, 1984). Upon this “deceptive” mating encounter, these cells will form super-assemblies of the Whi3 protein (termed “mnemon”, defined as a prion-like protein which retains information on past experiences; Reichert and Caudron, 2021). Since Whi3 normally arrests cell cycle progression (Garí et al., 2001), trapping Whi3 in such mnemons results in cells that require higher pheromone levels for cell cycle arrest and shmoo development (Caudron and Barral, 2013). This type of HDB is not passed on over generations because the large protein complexes stay within the mother cell during the budding process (asymmetric protein inheritance, Figure 2). Hence, while the mother cells will be less likely to form a shmoo after a few deceptive mating encounters, daughter cells are naïve and will shmoo upon detection of pheromones.

Interestingly, mnemons are not the sole source of protein-based HDB in mating behavior in *S. cerevisiae*. Pheromone detection activates the cell-cycle inhibitor Far1 (Chang and Herskowitz, 1990; Peter et al., 1993; Peter and Herskowitz, 1994), which acts as an amplifier of the pheromone signal. The cytoplasmic pool of Far1 is not degraded due to anchoring and complex formation with Cdc24. The higher the pheromone concentration at first exposure, the longer these cells will stay arrested upon second exposure, regardless of the second pheromone concentration (Atay and Skotheim, 2017). In contrast to the Whi3 mnemons, the Far1 pool is partly transferred to the daughter cells (symmetric protein inheritance, Figure 2), allowing transgenerational HDB.

Another example of putative protein-based HDB was recently described for HDB of past stress exposures in yeast. Yeast cells exposed to a mild stress display increased tolerance to subsequent, more severe stress exposures (Berry and Gasch, 2008; Guan et al., 2012). Studies suggest that inheritance of specific proteins underly at least some of this HDB. A recent study also implicated stress-activated RNA-protein granules in the HDB to stress exposure, although it remains to be investigated exactly how these granules contribute to HDB (Jiang et al., 2020; Escalante and Gasch, 2021).

While some of these protein-dependent HDB examples are reminiscent of the phenomenon of prions, there is some debate on the actual involvement of prions in HDB. Many prions are detrimental to yeast cells (Wickner, 2011; Wickner et al., 2011; Kelly et al., 2012), and yeast prions are extremely rarely found in nature (Nakayashiki et al., 2005; Halfmann et al., 2012; Kelly et al., 2012). On the other hand, prions can be inherited from mother to daughter cell where they display similar phenotypes over many generations and can even lead to beneficial phenotypes in specific environments, alluding to the potential involvement of prions in HDB (Alberti et al., 2009; Brown and Lindquist, 2009; Chernova et al., 2014; Chakravarty and Jarosz, 2018). Notably, sometimes also a distinction can be made between protein-dependent HDB and possible involvement of prions in HDB. Prion formation is the consequence of a conformational change that makes the proteins non-functional, i.e., they become incompetent to perform their normal cellular function. In some cases of protein-dependent HDB examples, the proteins involved are also incapable of performing their normal cellular function (because they are trapped in superassemblies, mnemons, for example), reminiscent of the phenomenon of prions. On the other hand, in other cases of protein-dependent HDB, such as galactose HDB, it is rather the total protein level that appears to be responsible (but see also below for other mechanisms involved in HDB in fluctuating carbon environments).

### Metabolic state

Multiple studies support the importance of cytoplasmic factor(s) produced during a specific environment as a mechanism for HDB. Although protein inheritance seems to be a recurring theme, other, broader, mechanisms have also been suggested. Indeed, studies on the previously mentioned galactose HDB revealed that, although inheritance of galactose metabolism-specific proteins aids the HDB effect, other factors and biomolecules such as mitochondria and ATP, and perhaps even the general metabolic state of a cell, may also contribute to HDB (Figure 2; Perez-Samper et al., 2018; Jariani et al., 2020). This will be further discussed in the next section.

## Case study: History-dependent behavior in fluctuating carbon environments

Glucose is the preferred carbon source of *S. cerevisiae* and presence of glucose blocks consumption of other carbon sources by repressing genes needed for metabolism of these alternative carbon sources (so-called glucose catabolite repression; Gancedo, 1998). Catabolite repression also represses genes required for respiration, even in the presence of oxygen. This explains why in high glucose concentrations, *S. cerevisiae* cells ferment glucose even in the presence of oxygen, when the more energy-efficient respiratory metabolism would be possible (16–18 ATP molecules/glucose *via* respiration versus only 2 ATP/glucose *via* fermentation). This preference for fermentation over respiration is referred to as the Crabtree effect (Crabtree, 1929). As outlined below, several recent studies point at a critical role for respiration and Crabtree repression in HDB during carbon source shifts.

Transition from glucose to a non-preferred carbon source results in a lag phase, and previous exposures to non-preferred carbon sources lead to reduced lag phase lengths. This makes the duration of the lag phase an ideal readout for HDB (Figure 1B). Moreover, HDB (and lag phase length) can be studied at both population level as well as individual cell level in single-celled organisms such as *S. cerevisiae*. Several mechanisms have already been proposed for HDB in fluctuating carbon environments. As described in the previous sections, such HDB is often contributed to the inheritance of key metabolic proteins (Stockwell et al., 2015). However, even though a correlation between Gal protein inheritance and HDB has been observed, studies found that exposure to other environments (such as maltose or glycerol) also resulted in HDB on galactose, without Gal protein induction (Cerulus et al., 2018). In fact, while induction of these key metabolic genes is crucial for cells to resume fast growth on alternative carbon sources, actual levels of these metabolic proteins are not the major determinants of HDB observed after carbon source shifts (Cerulus et al., 2018). This indicated that additional mechanisms are at play which may represent the key bottleneck for restarting growth.

To determine the mechanisms underlying HDB in glucose-maltose shifts, Cerulus et al. (2018) performed two genome-wide screens. Using BarSeq of the yeast deletion collection, they identified genes involved in respiration and mitochondrial function to be essential for HDB. Transcriptome analysis demonstrated that genes in respiration-linked pathways, such as the TCA cycle and respiratory chain, are induced during glucose-maltose shifts. Importantly, activation of these genes precedes the induction of genes that are specifically required for maltose metabolism. Additional experiments showed that changing respiratory activity also changed HDB, with reduced respiratory activity resulting in longer lag phases. Similar results were obtained when cells were shifted from glucose to galactose (Perez-Samper et al., 2018). Moreover, differences in the respiratory capacity of natural yeast strains also inversely correlate with the different lag times that are typically observed between natural yeast strains (Wang et al., 2015; Perez-Samper et al., 2018), further suggesting a central role for respiration in HDB, when using lag time as a proxy for HDB. Specifically, it is hypothesized that cells need to induce respiration to efficiently grow on non-preferred carbon sources, and perdurance and inheritance of proteins and other biomolecules or complexes that are required for respiration enable a faster transitioning to respiratory metabolism upon a second shift.

Interestingly, isogenic yeast populations often also show cellular heterogeneity in their HDB, with individual cells in an isogenic population displaying vastly different lag times. For example, when shifting from glucose to maltose, some yeast cells resume growth within 5 h, while others take more than 20 h, and some cells even never resume growth (New et al., 2014; Cerulus et al., 2018). This suggests that HDB could be further modulated by heterogeneity across single cells. Single-cell RNA seq allowed to investigate the possible molecular mechanism underlying HDB heterogeneity in the switch from glucose to maltose (Jariani et al., 2020). These results showed heterogeneity in gene expression between individual cells, and demonstrated that, similar to what was observed at population level, individual yeast cells also induce respiratory genes prior to escaping the lag, and cells failing to induce these genes fail to resume growth.

Why would respiratory activity be such a determining factor of HDB for carbon source changes? When cells are transferred from glucose to a non-preferred carbon source, they experience a drop in ATP levels, since there no longer is a glucose flux that allows for ATP production (Perez-Samper et al., 2018; Jariani et al., 2020). This drop in ATP levels coincides with growth arrest. Only cells that manage to restore ATP levels can resume growth, and respiration would allow for this restoration of energy.

Taken together, these results indicate the importance of respiration for HDB. How to then explain the observation that cells that have grown for longer times on glucose are much slower in restarting growth on alternative carbon sources compared to cells exposed to glucose for a shorter time (Cerulus et al., 2018)? Cells that have grown for extended times on glucose gradually repress respiration in favor of fermentation, and experience difficulties to re-activate respiratory metabolism because this requires synthesis and assembly of several complex molecules and cellular structures. However, cells that have recently been exposed to a shift that required them to activate respiration may still have some of these molecules and structures, allowing them to re-activate respiration much more easily. Similarly, stochastic differences in the basal level of respiratory activity between cells growing on glucose may explain the observed variability in the response between cells in a population.

Note that this mechanism of HDB is not specific to a particular carbon source. Instead, it could be more general, with cells ‘remembering’ growing on any alternative carbon source that does not repress respiration as much as glucose does. This also raises the question whether a cell’s metabolic state would also influence other forms of HDB.

## Evolutionary implications of history-dependent behavior

The ability of cells to exhibit a response depending on past experiences raises several questions about the possible implications as well as evolutionary causes of this HDB. Is HDB beneficial for cells? Would there be specific conditions selecting for HDB? Are there any trade-offs associated with exhibiting HDB? Is stochastic variation in HDB between isogenic cells a form of bet-hedging that has been selected for, or merely a consequence of biological noise?

Let us first consider the question of how long it should take for a cell to respond to changes in its environment. Naively, one would expect that a quick response is always better than a slow one. This would also imply that having a strong HDB (fast response to recurring condition) would always be beneficial, because it ensures optimal fitness in the new environment. However, inducing an appropriate response requires time and energy, with the risk that this response might not be useful or even detrimental when the original environment returns or when the environment quickly changes to yet another state. In these cases, theory has shown that either a slow response or a diversified response might be a much better strategy to optimize fitness over the long term (Kussell and Leibler, 2005; Botero et al., 2015; Ogura et al., 2017; Fritz et al., 2019).

Both theoretical and experimental studies have suggested that HDB of past stressful environments could be a relevant survival strategy for the population (Jablonka et al., 1995; Kussell and Leibler, 2005; Mitchell and Pilpel, 2011; Lambert and Kussell, 2014; Sivak and Thomson, 2014; Skanata and Kussell, 2016; Kronholm, 2022). Mitchell and Pilpel developed a model to simulate the fitness landscape across different conditions (Mitchell and Pilpel, 2011). They identified specific parameters that could lead to a fitness advantage for cells displaying HDB in an environment where there is a typical temporal order of environmental change. Under these conditions, cells can use the appearance of one specific condition as a predictive cue for the likely arrival of another condition. Their model suggests that such anticipatory behavior can be beneficial under stressful conditions, even in environments with varying time between the different conditions.

Multiple studies suggest that the ability to adapt to changing environments comes at a fitness cost for growth in stable environments (New et al., 2014; Venturelli et al., 2015; Bagamery et al., 2020). For example, evolving yeast cells in alternating glucose and maltose conditions resulted in cells displaying a shorter lag phase when transitioning from glucose to maltose (New et al., 2014). While these cells displayed strong HDB, they also displayed a reduced growth rate on glucose. This suggests that strong HDB and the resulting fast transitions could lead to fitness trade-offs in a constant environment. In a separate study, Venturelli et al. (2015) observed a heterogeneous response of genetically identical yeast cells to a specific combinatorial environment of glucose and galactose. Part of the population already induced *GAL* genes before the switch to galactose, and the fraction of these cells in the population depended on the sugar concentrations used. This early induction incurred a fitness benefit (shorter lag) when cells ran out of glucose and needed to switch to galactose, but also resulted in cells growing slower on glucose. Interestingly, natural *S. cerevisiae* isolates grown on a mix of glucose and galactose display varying lag phase length when switching from glucose to galactose, and this could be linked to differences in *GAL* gene induction timing prior to glucose exhaustion (Wang et al., 2015). Also in these natural strains, this “preparation” for glucose depletion has an immediate fitness cost and results in slower growth on glucose, but a delayed benefit since it results in a shorter lag and hence faster switching to galactose consumption. In other words, cells might continuously need to balance immediate fitness with future fitness benefits. Pre-emptive induction of metabolic programs could be a general microbial strategy to prepare for depletion of (preferred) nutrients in mixed (nutrient) environments (New et al., 2014; Venturelli et al., 2015; Wang et al., 2015).

Heterogeneity in the metabolic state of different cells within an isogenic population could be a diversified bet-hedging strategy that allows genetic lineages to balance fitness in the current environment with the ability to continue growth upon a sudden environmental change (Arnoldini et al., 2012; Müller et al., 2013). Research shows that there is variation in the proportion of these fractions in natural strains, hinting at the fact that natural ecologies might select for specific ratios depending on the exact environmental conditions (Bagamery et al., 2020). Interestingly, stochastic switching behavior is an inheritable phenotypic trait. Mother cells and their respective daughter cells synchronously switch between phenotypic states for long time periods (Kaufmann et al., 2007), indicating that population heterogeneity can be passed on and selected for. Using a theoretical model, Xue and Leibler (2016) showed that positive feedback that enhances the probability of the offspring to express the same phenotype as the parent can result in “evolutionary learning” of adaptation to variable environments. Such transgenerational memory can help adjust the level of heterogeneity within a population, and also allows HDB to last for multiple generations, instead of being restricted to the cells that originally encountered the environmental change. Models also predict that fast switching rates between phenotypes are more favorable in rapidly changing environments, while slow switching rates are more favorable in slowly changing environments (Lachmann and Jablonka, 1996; Kussell et al., 2005; Kussell and Leibler, 2005; Acar et al., 2008; Arnoldini et al., 2012). Importantly, mathematical models suggest that the fitness effect of a fraction of maladapted cells in a population is much smaller than one would intuitively predict (Cerulus et al., 2016). Hence, population heterogeneity could be useful to always have a fraction of cells prepared for a possible change in the environment.

One caveat of some of the published work is that theoretical studies often model extreme HDB and stochasticity, sometimes combined with extreme cases of environmental regimes. For example, models consider extreme switching rates without considering intermediate rates, and without any notion about switching rates that are observed in nature (Ardaševa et al., 2020). Moreover, the environmental changes considered are often sudden changes, while gradual changes likely occur frequently. While these theoretical studies have yielded valuable theoretical concepts, it is unclear how relevant these models are for actual microbial behavior since there is little experimental support for naturally occurring cases and conditions. The growing body of experimental evolution data showing clear trade-offs between evolving HDB and other phenotypes (New et al., 2014; Van den Bergh et al., 2016; Boyer et al., 2021) in principle allows simulating the evolutionary trajectory of HDB in more realistic environments (Van den Bergh et al., 2016; Tikhonov et al., 2020).

An example of an adaptive dynamics model (also called “evolutionary stable strategy modelling” in the field of theoretical biology (Dieckmann and Ferrière, 2004; Claessen et al., 2007; McGill and Brown, 2007; Salathé et al., 2009; Arnoldini et al., 2012; Brännström et al., 2013; Rubin and Doebeli, 2017)) to investigate optimal responses in variable environments is a study by Van den Bergh et al. (2016) on the evolution of persistence in bacteria. Their model demonstrates that the evolutionary stable state of persistence is not only tuned to the frequency at which antibiotics are administered but also to the duration of non-treatment periods. They show that the persistence duration needs to last sufficiently long to survive the antibiotic treatment, but at the same time also be short enough to grow at the fullest extent in conditions without antibiotics.

Knowledge on trade-offs makes “adaptive dynamics” an ideal modelling method to investigate HDB. Another possibility of modelling framework that can be applied to HDB is individual based models (DeAngelis and Mooij, 2005; Hellweger and Bucci, 2009; Hellweger et al., 2016). In this type of framework, each cell starts with the same parameter value (examples of possible parameters: lag duration, leaky gene expression, growth rate, internal metabolic change, protein turnover/degradation, …). These parameter values can then change with small but random increments for each cell individually to simulate evolution, where the most favorable changes will be selected for. This is in contrast with adaptive dynamics, where (different) fixed parameter values are taken for different cells and these cells are then competed against each other. This allows to see which cell (which specific parameter value) displays the highest fitness, allowing to investigate how well a cell with specific parameter values can invade an existing population. Since individual based models allow simulating evolution to some degree, they also in principle would make it possible to see in which direction a phenotype would evolve. However, to see if there is a consistent evolutionary trajectory or to see more general phenomena the simulation needs to be ran many times. Moreover, individual based models for micro-organisms are computationally more expensive since the population consists of millions or even billions of cells that need to be tracked individually.

Evolutionary models can be applied to check the feasibility and evolvability of a specific phenotype. One interesting phenomenon/mechanism from an evolutionary context is leaky gene expression, where gene expression persists for a while after its external inducer has already disappeared, resulting in cell-to-cell heterogeneity in HDB. If this causative environment would return, then these cells would be able to adapt/respond faster. This also raises the intriguing possibility that expression levels are perhaps evolutionary tuned to not only enable growth in a specific environment, but also to allow for HDB. Using an evolutionary model would allow us to investigate which are the ideal conditions for leaky gene expression for a range of environmental changes. Gene expression levels, persistence of gene expression, protein lifetime, metabolic constraints,…: all are possible parameters in an adaptive dynamics - or individual based model to gain insight into the evolutionary trajectories and fitness optima of HDB in fluctuating environments. Using these parameters in an adaptive dynamics model would allow computing a fitness landscape in a specific environment (Waxman and Gavrilets, 2005), whereas an individual based model would teach us what a “realistic” evolutionary trajectory would be for the evolution of leaky gene expression, and to which parameter optima the population will converge.

Accurate modeling remains a challenge, for example because details on gene expression dynamics, protein lifetimes and the trade-offs between HDB and other phenotypes (e.g. growth rate) remain difficult to measure (Dieckmann and Ferrière, 2004; Bowers et al., 2005). Determining fitness in changing environments is not straightforward and requires quantification of the long-term fitness advantage. Additionally, determining evolutionary trajectories for multidimensional traits such as antibiotic resistance and persistence remains difficult and has been much less explored. An alternative approach to modelling would be to investigate the evolution of HDB under various regimes in laboratory evolution experiments, since this would allow to determine, without *a priori* assumptions, if and what forms of HDB could evolve under specific environmental conditions.

Finally, whether or not HDB is a consequence of other mechanisms or itself is a result of adaptation is difficult to distinguish. A key factor here might be the predictability of the environmental change cells are faced with. Adaptive behavior can more easily arise when the environment changes in a predictable manner. An example of such an evolutionary tuned behavior is the anticipatory behavior of the gene regulation of *E. coli* in the mammalian gut (Mitchell et al., 2009), as discussed in the introduction. On the other hand, for microbial phenotypic heterogeneity, such as is observed for bet-hedging strategies, it is much harder to determine if this is specifically selected for or perhaps merely a “simple” consequence of internal mechanisms, such as noisy gene expression.

## Discussion and future perspectives

This review focused on HDB in the context of past environmental conditions. Apart from extracellular changes, also internal (stochastic) fluctuations can influence cellular behavior, and this cellular behavior can also display HDB. Norman et al. (2013) for example studied the transition between motile-unicellular and sessile-multicellular (chained) states in *Bacillus subtilis* in a constant environment. They observed a critical difference in switching between these states: a motile cell switched independently of its history (in other words, the probability of chaining is the same, whether a cell has been motile for 1 or 100 generations), whereas sessile “chains” displayed tightly tuned transitions, indicating memory of the cell’s own state in the latter case. This switching occurred in a constant environment and hence is influenced by internal cellular fluctuations. A follow-up study from the same group revealed that switching from a unicellular to multicellular state can be explained by stochastic fluctuations in the interaction between two proteins required for switching (Lord et al., 2019).

One hypothesis put forward by the authors is that this type of HDB could underly the earliest steps for multicellularity. HDB for cells in the chained state due to these stochastic fluctuations could give cells in a population a trial period for multicellularity, with external signals such as growth-related stresses or the presence of a desirable niche ultimately influencing long-term commitment to the chained state.

Despite much research on HDB, many questions still remain. Most importantly, while several examples and mechanisms underlying HDB have been described, the relevance of HDB in natural settings remains largely unknown. To date, almost all studies on HDB have been performed under controlled laboratory conditions. Moreover, the experimental conditions, including the environmental regime and the genetic background of the organisms, often differ significantly between studies. This makes it difficult to compare results and prevents drawing conclusions on how prevalent and important HDB is in natural settings. For example, if and how natural ecologies support and select for history-dependent behavior, how prevalent this behavior is in nature, or whether specific settings select for specific HDB mechanisms. Our understanding is perhaps mainly hampered by our limited knowledge of microbial ecology, including knowledge about dynamics of natural environmental fluctuations and ecosystems consisting of multiple species. This also makes it difficult to include and integrate relevant ecological parameters in models that are looking into effects and possible trade-offs associated with HDB. Additionally, experiments are typically carried out using a population of isogenic cells of a specific species, whereas in nature, microbes do not live in isolation but rather in complex communities. Interspecies and interstrain communication could potentially also affect microbial behavior, including HDB.

Apart from its potential relevance in natural settings, HDB, and in particular history-dependent changes in the lag phase, are relevant for biotechnological and industrial applications. In many of these applications, cells are growing in complex media under stressful conditions and need to switch between nutrients over time. During this switch, growth speed is drastically reduced and this often leads to stuck or sluggish fermentations, resulting in significant economic losses. A better understanding of the factors determining HDB and the underlying mechanisms could potentially help reduce the frequency of these stuck fermentations (Alexandre and Charpentier, 1998; Verstrepen et al., 2004).

Interestingly, multiple studies have observed differences in HDB between different natural yeast strains, with some strains resuming growth much faster after reappearance of a specific environment than others (Wang et al., 2015; Perez-Samper et al., 2018). This suggests the existence of genetic factors underlying natural variation in lag phase (and hence HDB). Identification of these genetic factors would be a major step forward for our understanding of HDB. The different levels of HDB observed between different genetic backgrounds also make it tempting to speculate that this could be due to differences in regulatory mechanisms between strains, with some strains perhaps showing a less stringent catabolite repression for example.

It is tempting to speculate that other major metabolic transitions, comparable to the transition from fermentation to respiration in HDB specific for carbon sources as discussed in the case study of this review, could govern HDB in other cell types for other fluctuating environments. This is especially tantalizing since it seems likely that many cases of (nutritional) environmental fluctuations also impact ATP levels and would require metabolic transitions to restore ATP levels.

## Author contributions

All authors listed have made a substantial, direct, and intellectual contribution to the work and approved it for publication.

## Funding

Original research performed in the lab of KJV is supported by the European Research Council (ERC CoG682009), FWO, KU Leuven (C1 project No. 3E170455) and VIB. LV acknowledges FWO SB Grant SB/1S07117N. TW acknowledges support from KU Leuven (C1 project No. 3E170455).

## Conflict of interest

The authors declare that the research was conducted in the absence of any commercial or financial relationships that could be construed as a potential conflict of interest.

## Publisher’s note

All claims expressed in this article are solely those of the authors and do not necessarily represent those of their affiliated organizations, or those of the publisher, the editors and the reviewers. Any product that may be evaluated in this article, or claim that may be made by its manufacturer, is not guaranteed or endorsed by the publisher.
